# Immune Checkpoint Inhibitors Plus an Anti-VEGF Antibody as the First-Line Treatment for Unresectable Hepatocellular Carcinoma: A Network Meta-Analysis and Cost-Effectiveness Analysis

**DOI:** 10.3389/fphar.2022.891008

**Published:** 2022-06-01

**Authors:** Lu Li, Shilei Yang, Yanwei Chen, Li Tian, Ying He, Bin Wu, Deshi Dong

**Affiliations:** ^1^ Department of Pharmacy, First Affiliated Hospital of Dalian Medical University, Dalian, China; ^2^ Department of Pharmacy, Ren Ji Hospital, Shanghai Jiao Tong University School of Medicine, Shanghai, China

**Keywords:** network meta-analysis, unresectable hepatocellular carcinoma, HCC, cost-effectiveness, immune checkpoint inhibitors plus an anti-VEGF antibody

## Abstract

**Background:** Sintilimab + a bevacizumab biosimilar (IBI305) (SB) and atezolizumab + bevacizumab (AB) have been approved for the treatment of unresectable hepatocellular carcinoma (HCC). At present, oncologists and their patients remain indecisive on their preferred treatment regime. Therefore, assessing their efficacy *via* a network meta-analysis and determining their comparative cost-effectiveness is necessary.

**Objective:** To evaluate the cost-effectiveness of SB and AB compared with sorafenib alone for the treatment of unresectable HCC.

**Materials and Methods:** The data used in our analysis were obtained from patients in ORIENT-32 and IMbrave150 phase III randomized clinical trials. A Bayesian network meta-analysis and cost-effectiveness analysis that included 1,072 patients were performed in this study. A partitioned survival model was applied to the patients with unresectable HCC. The model was designed with a 15-year time horizon, 1-month cycle, and 5% discount rate for costs and outcomes. In China, an incremental cost-effectiveness ratio (ICER) value of less than $33,500 (three times the GDP per capita in 2020) per quality-adjusted life-year (QALY) is considered cost-effective. The influence of parameter uncertainty on the results was verified by one-way deterministic sensitivity analysis and probability sensitivity analysis. Furthermore, scenario analyses of the patient assistance program (PAP) were conducted to explore the cost-effectiveness of SB and AB.

**Results:** For the model of 1,072 patients, treatment with SB produced an additional 0.617 QALYs compared with sorafenib, resulting in an ICER of $39,766.86/QALY. Similarly, treatment with AB produced an additional 0.596 QALYs compared with sorafenib, resulting in an ICER of $103,037.66/QALY. The probability sensitivity analysis showed that when the willingness-to-pay (WTP) threshold was $33,500/QALY, the cost-effectiveness of SB and AB was 15.4 and 0.4%, respectively. However, in the scenario analyses, the probability of SB and AB regimens being cost-effective was 65.4 and 15.8%, respectively, at a WTP of $33,500/QALY.

**Conclusion:** The findings from our study showed that sintilimab + a bevacizumab biosimilar is a cost-effective regimen compared with sorafenib as the first-line therapy for unresectable HCC in China at a $33,500 WTP threshold if sintilimab PAP is considered. However, the atezolizumab + bevacizumab regimen is not cost-effective whether atezolizumab PAP is considered or not.

## 1 Introduction

Primary liver cancer is the fourth leading cause of cancer-related death worldwide. In 2018, an estimated 781,631 deaths occurred globally and 368,960 deaths occurred in China, accounting for approximately 50% of the deaths worldwide (Bray et al., 2018; Valery et al., 2018). Hepatocellular carcinoma (HCC) accounts for approximately 80% of liver cancers and has a great impact on society and the economy (Perz et al., 2006). Unfortunately, only 30–40% of patients are diagnosed at an early stage and receive effective treatment (Forner et al., 2018). Over the past decade, new therapeutics have significantly improved the resectability of liver metastases and prolonged survival in advanced unresectable HCC. Such therapies include sorafenib and lenvatinib as first-line treatments, and regorafenib, cabozantinib, and ramucirumab as second-line treatments (Kong et al., 2020).

Liver cancer is often complicated by liver inflammation that exacerbates this condition. The combination of anti-PD-1 and anti-PD-L1 monotherapy or in combination with molecular targeted therapy, other immunomodulators, or cytotoxic chemotherapy has contributed to the progress in this area (Finn et al., 2020a; Finn et al., 2020b; Yu et al., 2020; Yau et al., 2020; Ren et al., 2021; Yau et al., 2022). Reliable predictors of immune checkpoint inhibitor (ICI) response are essential to allow appropriate stratification and selection of HCC patients to obtain more benefits from immunotherapy (Rizzo and Ricci, 2022). Of these, combined anti-vascular endothelial growth factor (anti-VEGF) and immunotherapies are expected to resolve the issues associated with the immunosuppressive tumor microenvironment of HCC (Fukumura et al., 2018).

Two phase III clinical trials [ORIENT-32 (Ren et al., 2021) and IMbrave150 (Finn et al., 2020a)] have shown a survival advantage of ICIs combined with anti-VEGF therapy compared with the standard treatment (sorafenib) for unresectable HCC. In the ORIENT-32 trial, sintilimab + a bevacizumab biosimilar (IBI305) (SB) may improve the overall survival (OS) [hazard ratio (HR) 0.57, 95% confidence interval (CI) (0.43–0.75)], and the median progression-free survival (PFS) time of patients was 4.6 months. In the IMbrave150 trial, atezolizumab + bevacizumab (AB) led to a higher OS rate (HR 0.58, 95% CI (0.65–0.98)], and the median PFS time in AB was 6.8 months.

AB has been approved by the US Food and Drug Administration (FDA) and the China National Medical Products Administration (NMPA) for the up-front treatment of patients with unresectable or metastatic HCC on May 29, 2020, and October 28, 2020, respectively (Genentech, 2022; Roche, 2022). However, because the ORIENT-32 trial only recruited Chinese patients, the SB regimen was only approved by NMPA for the first-line treatment of patients with unresectable or metastatic HCC on June 25, 2021 (The drug approval, 2022) and no other countries approved this regimen.

Thus, ICIs combined with an anti-VEGF antibody opened a new age for the unresectable HCC. Hence, from the perspective of the Chinese healthcare system, we examined the cost-effectiveness of two schemes (SB and AB vs. sorafenib) in the first-line therapy of unresectable HCC.

## 2 Materials and Methods

The patient baseline characteristics of ORIENT-32 and the IMbrave 150 trials are given in Supplementary Table S1. The ORIENT-32 trial (NCT03794440) started in February 2019 and confirmed the efficacy and safety of SB in advanced or unresectable HCC. The IMbrave150 trial (NCT03434379) started in March 2018 and confirmed the efficacy and safety of AB in metastatic advanced or unresectable HCC.

In this study, we used the method of cost-effectiveness analysis (CEA). In the CEA, decision-making is based on an incremental analysis. An incremental analysis compares the costs and results of the intervention with those of the comparator. The intervention will become the strictly dominant treatment scheme when it has a lower cost and better outcome than the comparator. In contrast, the interventions will be strictly subordinated to the treatment scheme when it has a higher cost and poorer outcome compared with the comparator. In circumstances where the intervention treatment scheme has a higher cost and better outcome than the comparator, the incremental cost-effectiveness ratio (ICER), that is, the ratio of the difference in costs to the difference in outcomes between the two regimens, needs to be calculated. If the ICER is smaller than or equal to the threshold value, the intervention is a more cost-effective choice than the comparator; if the ICER is larger than the threshold value, the intervention is not a cost-effective choice compared with the comparator (Liu et al., 2020). Quality-adjusted life-years (QALYs) are recommended as indicators for the outcome measurement. The formula for ICER is as follows (Cai et al., 2019):

$$\mathrm{ICER}=\left(C_{A}-C_{B}\right)/\left(\mathrm{QAL}Y_{A}\mathrm{-QAL}Y_{B}\right)=\mathrm{\Delta C}/\mathrm{\Delta QALY},$$

where C_i_ and QALY_i_ represent the patient’s overall cost and effectiveness of treatment (i = A) or comparator (i = B).

### 2.1 Network Meta-Analysis

We searched PubMed, Embase, and the Cochrane Central Register of Controlled Trials (CENTRAL) for eligible publications, selecting manuscripts published up to June 25, 2021. The Clinicaltrials.gov database was also searched. The search terms used were, “Atezolizumab,” “Sintilimab,” “Pembrolizumab,” “Novoliumab,” “Camrelizumab,” “Durvalumab,” “Toripalimab,” “Tislelizumab,” and “Unresectable hepatocellular carcinoma” as medical subject keywords. The details of the filters are shown in Supplementary Figure S1. Multiple reports of the same clinical trial and trials which did not contain a control group, or those which were non-randomized or included other interventions, were excluded from this analysis.

We implemented the Bayesian network meta-analysis in R, version 4.0.5, with the package of “gemtc” to obtain the HRs for OS and PFS between SB, AB, and sorafenib. The pooled HRs for OS and PFS were used for the cost-effectiveness analysis. The risk of bias for the clinical trials was assessed using RevMan, version 5.4. Owing to the lack of data to assess inter-trial heterogeneity, we applied a fixed-effects model for the analysis (Su et al., 2020).

### 2.2 Model Structure

**FIGURE 1:**
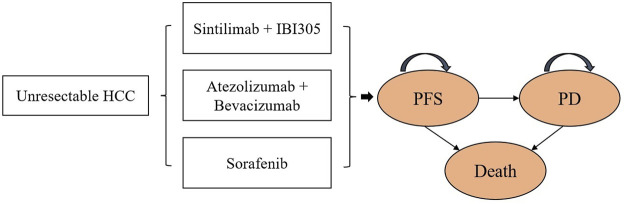
Partitioned survival model. HCC, unresectable hepatocellular carcinoma; PFS, progression-free survival; PD, progressive disease.

A partitioned survival model of unresectable HCC was exploited in Microsoft Excel to calculate the healthcare costs and health outcomes of the following three strategies: SB, AB, and sorafenib. The model included three health states: progression-free survival (PFS), progressive disease (PD), and death (Figure 1).

In the cost-effectiveness analysis, we compared the cost-effectiveness of SB and AB against sorafenib (reference strategy). The model cycle length was 1 month and the time horizon was 15 years. Both costs and utilities were discounted at a rate of 5% per year (Liu et al., 2020). We measured the overall costs, QALYs, life-years (LYs), and ICERs of the test therapies and references. The willingness-to-pay (WTP) threshold for China was $33,500 per QALY (three times the GDP per capita in 2020). The initial state is assumed to be PFS and death is assumed to be the absorbing state.

### 2.3 Efficacy Estimates

Efficacy should be based on the best available evidence. For newer drugs, clinical efficacy data from a randomized controlled trial (RCT) are preferred when available and applicable (Liu et al., 2020). The co-primary endpoints of ORIENT-32 and IMbrave150 were OS and PFS, respectively, as assessed by an independent review facility using Response Evaluation Criteria in Solid Tumors (RECIST) version 1.1 (Finn et al., 2020a; Ren et al., 2021). To construct the survival model, the GetData Graph Digitizer (version 2.26) was used to extract graphic data from the K–M curves (OS and PFS curves) of the two trials (ORIENT-32 and IMbrave150). Fitting of the parameter model requires time-event individual patient data (IPD) using the approach suggested by Guyot et al. (2012) By fitting the IPD, the parametric regression model method was chosen among the gamma, Gompertz, Weibull, exponential, log-normal, and log-logistic distributions, based on the Akaike information criterion (AIC) value. The reproduced digitized Kaplan–Meier (KM) curves are shown in Supplementary Figures S2, S3. We pooled the virtual IPD in the sorafenib arm of the two clinical trials and fitted the OS and PFS data by log-logistic and log-normal distributions according to the outcomes of the goodness of fit of the AIC statistic (Supplementary Figure S4). The final parametric model is shown in Supplementary Table S2. The model-fitted K–M curves are shown in Supplementary Figure S5.

### 2.4 Clinical Inputs

Based on the ORIENT-32 and IMbrave 150 trials, sorafenib was prescribed at a dose of 400 mg orally, twice daily (Finn et al., 2020a; Ren et al., 2021). Patients in the SB group received 200 mg of sintilimab and 15 mg/kg of IBI305 intravenously every 3 weeks, and tumor assessments were conducted by contrast magnetic resonance imaging (MRI) or computed tomography (CT) at the baseline and every 6 weeks until week 48, and then every 12 weeks (Ren et al., 2021). Patients in the AB group were administered 1,200 mg atezolizumab and 15 mg/kg bevacizumab intravenously every 3 weeks, and tumor assessments were assessed by MRI or CT at the baseline and every 6 weeks until week 54, and then every 9 weeks thereafter (Finn et al., 2020a). The drug dosages were calculated using an average weight of 60 kg (Wen et al., 2021). The SB and AB treatments were continued until unacceptable toxicity or disease progression occurred, or until 2 years of follow-up. Treatment with sorafenib was continued until unacceptable toxicity or disease progression was observed. The percentages of SB, AB, and sorafenib patients receiving second-line therapy were 29, 35, and 57%, respectively (Finn et al., 2020a; Ren et al., 2021). Regorafenib (a tyrosine kinase inhibitor) was approved as a second-line treatment for patients in whom first-line treatment was ineffective (Bruix et al., 2017).

The analysis included grade three or four adverse events (AEs) with greater clinical influence in the ORIENT-32 and IMbrave 150 trials: hypertension, proteinuria, nausea, thrombocytopenia, diarrhea, palmar-plantar erythrodysesthesia syndrome, increased aspartate aminotransferase, and increased alanine aminotransferase (Finn et al., 2020a; Ren et al., 2021).

### 2.5 Cost Inputs

In this study, we only considered direct medical costs, including the drug costs of sintilimab, atezolizumab, bevacizumab, and its similar, test costs, grade three or four AEs costs, follow-up costs, and subsequent costs after disease progression (Table 1) (Wu et al., 2012; Hou and Wu, 2020; Chinese drug, 2021; Wen et al., 2021). The drug costs were estimated from the local bid-winning price (Chinese drug, 2021). The incidence rates of major grade three or four AEs for different treatments are shown in Table 2. All costs were converted into US dollars using the exchange rate: $1 = ¥6.49.

**TABLE 1:** Input parameters of the model.

Parameter	Baseline value	Lower limit	Upper limit	Distribution	Source
Survival model of sorafenib					Finn et al. (2020a); Ren et al. (2021)
Log-logistic OS survival model	shape = 1.577	ND	ND	ND	Model fitting
	scale = 11.477				
Lognormal PFS survival model	meanlog = 1.2942	ND	ND	ND	Model fitting
	sdlog = 0.8621				
HR for OS (SB vs. sorafenib)	0.570	0.43	0.75	Lognormal	Network meta-analysis
HR for PFS (SB vs. sorafenib)	0.570	0.47	0.70	Lognormal	Network meta-analysis
HR for OS (AB vs. sorafenib)	0.580	0.42	0.79	Lognormal	Network meta-analysis
HR for PFS (AB vs. sorafenib)	0.600	0.47	0.76	Lognormal	Network meta-analysis
Drug cost (per month)					
Sintilimab	1168.16	934.52	1401.79	Gamma	Chinese drug (2021)
IBI305	2141.14	1712.91	2569.37	Gamma	Chinese drug (2021)
Atezolizumab	6738.57	5390.86	8086.29	Gamma	Chinese drug (2021)
Bevacizumab	2773.50	2218.80	3328.20	Gamma	Chinese drug (2021)
Sorafenib	1756.55				
Second-line therapy (per month)	2232.41	1785.93	2678.89	Gamma	Chinese drug (2021)
Percentage receiving second-line treatment					
SB group	29%	23.2%	34.8%	Beta	Ren et al. (2021)
AB group	35%	28%	42%	Beta	Finn et al. (2020a)
Sorafenib	57%	45.6%	68.4%	Beta	Finn et al. (2020a); Ren et al. (2021)
Test of AB (per month)	179.53	143.62	215.44	Gamma	Wen et al. (2021)
Test of SB (per month)	179.53	143.62	215.44	Gamma	Assumed equal to Test of AB (per month)
Test of sorafenib (per month)	167.56	134.05	201.07	Gamma	Wen et al. (2021)
Cost of follow-up in PFS (per month)	114.00	91.20	136.80	Gamma	Hou and Wu (2020)
Cost of follow-up in PD (per month)	210.00	168.00	252.00	Gamma	Hou and Wu (2020)
AEs cost (per event)					
Hypertension	16.50	13.20	19.80	Gamma	Wu et al. (2012)
Proteinuria	147.40	117.92	176.88	Gamma	Wu et al. (2012)
Nausea	56.60	45.28	67.92	Gamma	Wu et al. (2012)
Thrombocytopenia	4536.20	3628.96	5443.44	Gamma	Wu et al. (2012)
Diarrhea	188	150.4	225.6	Gamma	Hou and Wu (2020)
Palmar-plantar erythrodysesthesia syndrome	15	12	18	Gamma	Hou and Wu (2020)
AST	357.00	285.60	428.40	Gamma	Hou and Wu (2020)
ALT	357.00	285.60	428.40	Gamma	Hou and Wu (2020)
Health utility					
PFS state	0.76	0.61	0.91	Beta	Rabin and de Charro (2001)
PD state	0.68	0.54	0.82	Beta	Rabin and de Charro (2001)
Disutility due to AEs (grade ≥ 3)	0.16	0.13	0.19	Beta	Amdahl et al. (2016)
Death state	0.00	0.00	0.00	Beta	

OS, overall survival; PFS, progression-free survival; PD, progressive disease; HRs, hazard ratios; AEs, adverse events; ALT, alanine transaminase; AST, aspartate transaminase; SB, sintilimab plus a bevacizumab biosimilar (IBI305); AB, atezolizumab plus bevacizumab; ND, not determined.

**TABLE 2:** Incidence of adverse events.

Grade ≥3 AEs	SB	AB	Sorafenib
Hypertension	0.14	0.152	0.088
Proteinuria	0.05	0.03	0.012
Nausea	0.01	0.003	0.018
Thrombocytopenia	0.08	0.033	0.006
Diarrhea	0.02	0.018	0.038
Palmar-plantar erythrodysesthesia syndrome	0	0	0.103
AST	0.02	0.07	0.053
ALT	0.01	0.036	0.021

ALT, alanine transaminase; AST, aspartate transaminase; AEs, adverse events; SB, sintilimab plus a bevacizumab biosimilar (IBI305); AB, atezolizumab plus bevacizumab.

The costs of managing AEs per event in China were extracted from the published literature (Wu et al., 2012; Hou and Wu, 2020). We assumed that AEs occurred during the first model cycle.

Because of the high price of PD-1 and PD-L1, they are not affordable for many patients in China, and the sintilimab and atezolizumab patient assistance program (PAP) has been implemented for Chinese patients. In this program, sintilimab is paid for by the patients for the first two cycles, followed by donations for two cycles by Innovent Biologics (the producer of sintilimab); if the patients are still alive, they pay for the next five cycles, with the remaining cycles being funded by Innovent Biologics. Atezolizumab is paid for by the patients for the first two cycles, followed by donations for three cycles by F. Hoffmann-La Roche (the producer of atezolizumab); if the patients are still alive, they pay for the next two cycles, followed by donations for the remaining cycles by F. Hoffmann-La Roche. Therefore, the impact of PAP was evaluated using a scenario analysis.

### 2.6 Utilities Estimates

The utility score, ranging from 0 to 1, reflects the level of social functioning and physical, mental, and disease-related health states, where 0 represents the worst health status or death, and 1 represents the best health status. The utility estimates of PFS and PD states associated with advanced HCC were 0.76 and 0.68, respectively (Table 1) (Rabin and de Charro, 2001). Disutility values of grade three or four AEs were considered in the analysis (Table 1) (Amdahl et al., 2016). We assumed that AEs occurred during the first model cycle. Duration-adjusted disutility was subtracted from baseline PFS utility.

### 2.7 Sensitivity Analyses

In the sensitivity analysis, we conducted a series of uncertainty analyses of the variables listed in Table 1. The variables in this study included costs, utilities, hazard ratios (HR, from the network meta-analysis), proportion of patients, and probability.

One-way deterministic sensitivity analyses (DSAs) were performed by varying a single input to assess the robustness of the model results. The model parameters were varied by 95% CI if such information was reported in the source or varied by ± 20% from the base case values if the information was unavailable (Table 1) (Wen et al., 2021).

Probabilistic sensitivity analysis (PSA) was implemented using 1,000 Monte Carlo simulations. In each iteration, the model parameters were randomly extracted from the prescriptive distributions. The log-normal distribution was set for the variables of hazard ratio parameters, gamma distribution was set for the variables of cost parameters, and beta distribution was set for variables such as proportion of patients, probability, and utility value. The results are presented as a cost-effectiveness acceptability curve (CEAC).

In addition, one-way DSA and PSA were used to assess PAP scenarios.

## 3 Results

### 3.1 Network Meta-Analysis

Through a database search, 296 records were screened, and two phase III randomized clinical trials (ORIENT-32 and IMbrave150) with 1,072 patients were included in the network meta-analysis. A model schematic for the network meta-analysis is shown in Supplementary Figure S6. In the ORIENT-32 trial, 571 patients were administered SB (N = 380) or sorafenib (N = 191); in the IMbrave150 trial, 501 patients were administered AB (N = 336) or sorafenib (N = 165). The risk of bias is shown in Supplementary Figure S7. From the indirect comparisons of the network meta-analysis, both SB (HR 0.57, 95% CI, 0.43–0.75) and AB (HR 0.58, 95% CI, 0.42–0.79) could lead to great improvements in OS compared with sorafenib-related survival. The HRs for PFS of SB and AB, when compared with the sorafenib treatment, were 0.57 (95% CI, 0.47–0.70) and 0.60 (95% CI, 0.47–0.76), respectively.

### 3.2 Cost-Effectiveness Analysis

#### 3.2.1 Base-Case Analyses

For the model of 1,072 patients, SB treatment produced an additional 0.617 QALYs compared with sorafenib, resulting in an ICER of $39,766.86/QALY, and AB treatment produced an additional 0.596 QALYs compared with sorafenib, resulting in an ICER of $103,037.66/QALY (Table 3).

**TABLE 3:** Results of the base-case analysis.

Strategy	Cost ($)	LYs	QALYs	ICER
Sorafenib	18,567.66	1.59	1.11	
SB	43,109.99	2.47	1.73	39,766.86
AB	79,965.01	2.45	1.71	103,037.66

SB, sintilimab plus a bevacizumab biosimilar (IBI305); AB, atezolizumab plus bevacizumab; QALYs, quality-adjusted life years; LYs, life-years; ICER, incremental cost-effectiveness ratio.

#### 3.2.2 Sensitivity Analyses

**FIGURE 2:**
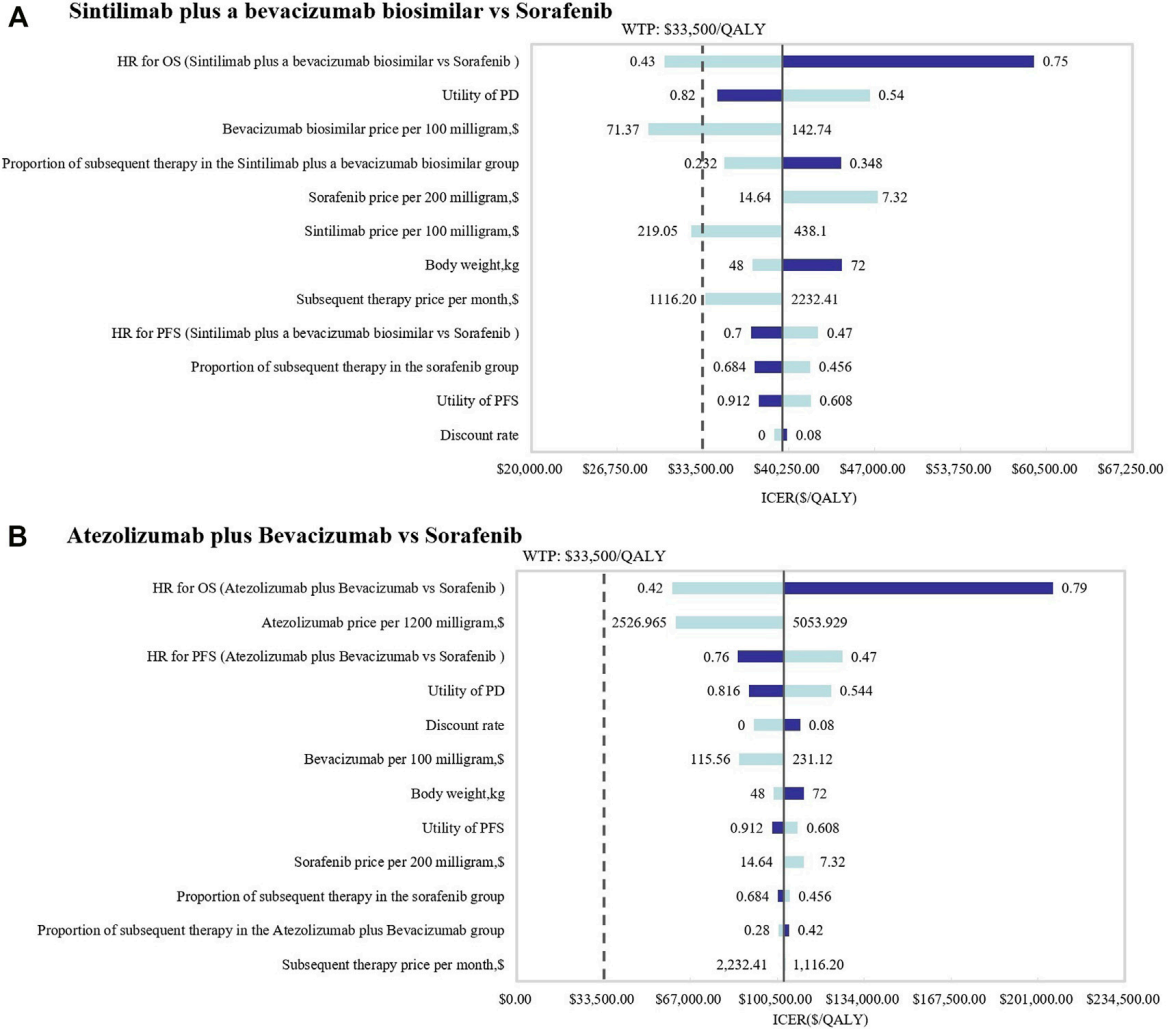
Tornado diagrams of one-way deterministic sensitivity analyses. One-way deterministic sensitivity analyses of **(A)** SB and **(B)** AB in comparison with sorafenib.

In this study, the results shown in the tornado diagram are the ICER values (Figure 2). The results indicated that the HRs of OS for both SB and AB regimens against sorafenib were the most sensitive parameters, and consequently, these had the most prominent impact on ICERs. When comparing SB with sorafenib, the results were also sensitive to the utility of PD and the price of the bevacizumab biosimilar, while the HRs of PFS and the price of atezolizumab were sensitive when AB was compared with sorafenib. As a result, the ICER value of SB versus sorafenib was less than the WTP threshold of $33,500 per additional QALY when the lower boundary of the HR (0.43) for OS was used or when the price of bevacizumab biosimilar and sintilimab was discounted by 50%. However, regardless of how the parameters changed, the ICER value of AB versus sorafenib therapy was not within the WTP ($33,500/QALY) threshold.

**FIGURE 3:**
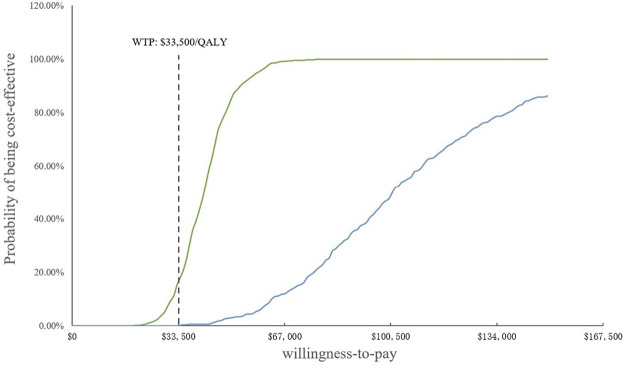
Cost-effectiveness acceptability curves. WTP, willingness-to-pay; QALY, quality-adjusted life year.

**FIGURE 4:**
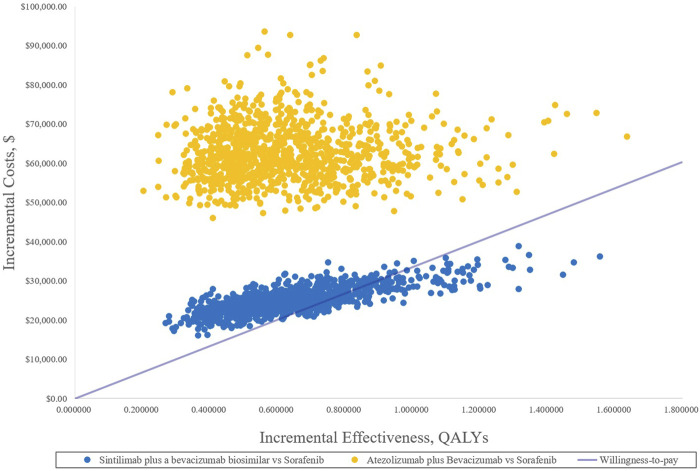
Incremental cost-effectiveness scatterplots. QALYs, quality-adjusted life years.

In the PSA, CEAC (Figure 3) showed that the probability of SB therapy being cost-effective was 16% compared with sorafenib at a WTP threshold of $33,500/QALY, and the corresponding probability of AB was less than 1% when compared with sorafenib. The incremental cost-effectiveness scatterplot is shown in Figure 4.

### 3.3 Scenario Analysis

**FIGURE 5:**
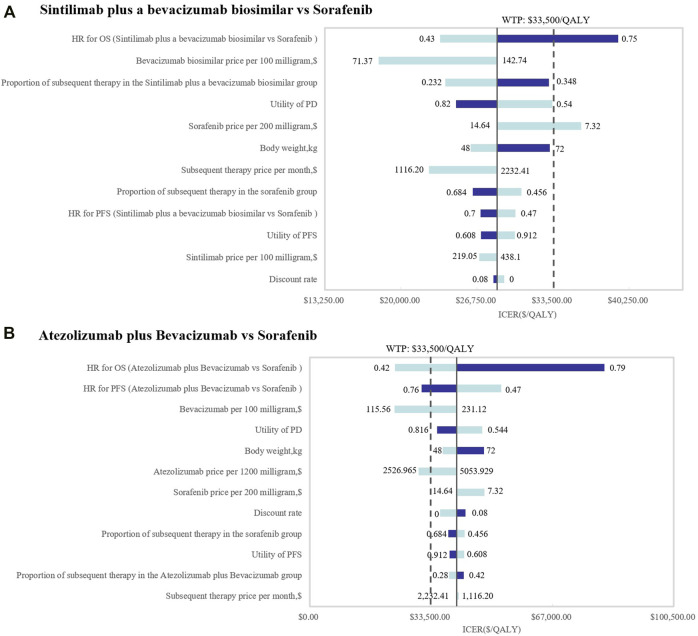
Tornado diagrams of the scenario analysis. One-way deterministic sensitivity analyses of **(A)** SB and **(B)** AB in comparison with sorafenib in the scenario analysis. OS, overall survival; PFS, progression-free survival; HR, hazard ratios; PD, progressive disease; ICER, incremental cost-effectiveness ratio; SB, sintilimab plus a bevacizumab biosimilar (IBI305); AB, atezolizumab plus bevacizumab; WTP, willingness-to-pay; QALY, quality-adjusted life year.

**FIGURE 6:**
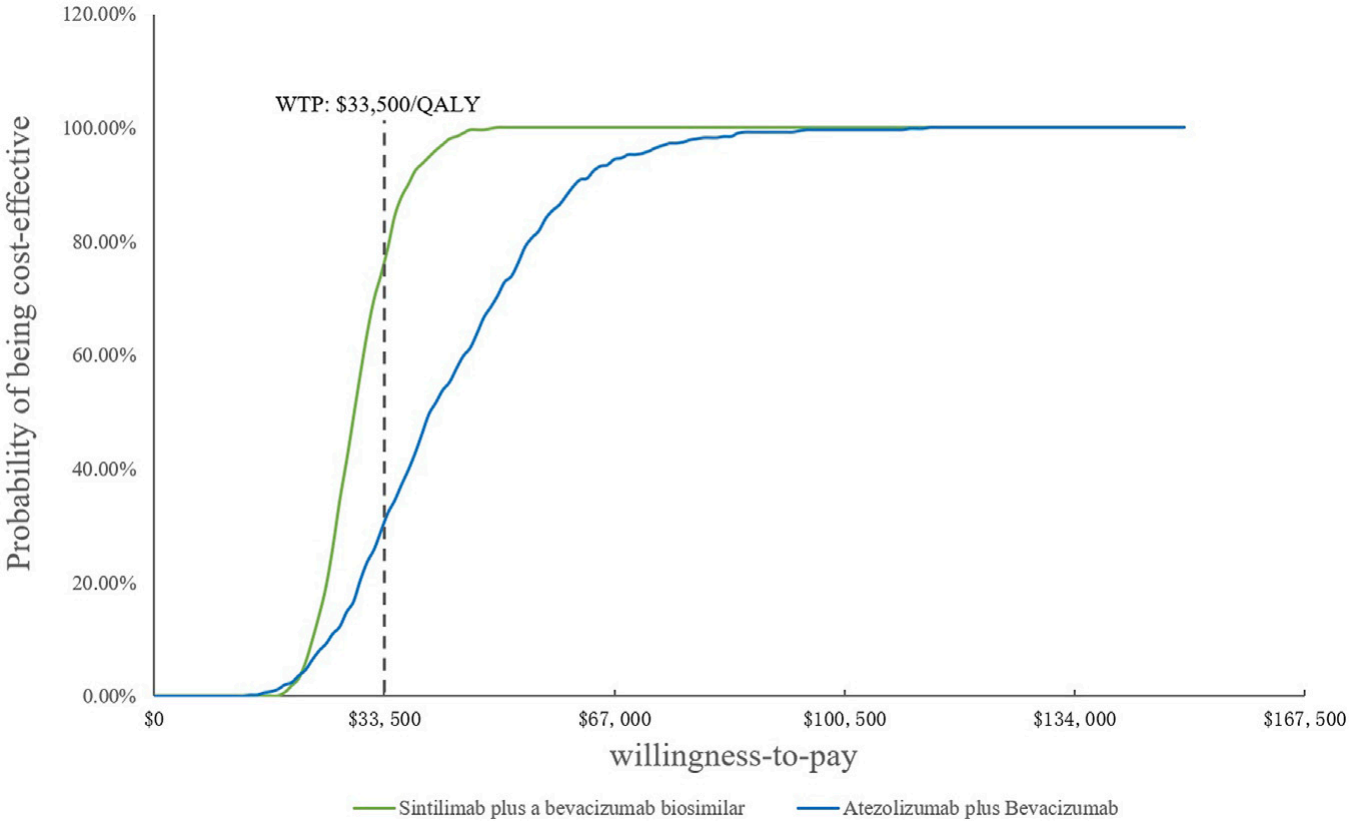
Cost-effectiveness acceptability curves of the scenario analysis. WTP, willingness-to-pay; QALY, quality-adjusted life year.

**FIGURE 7:**
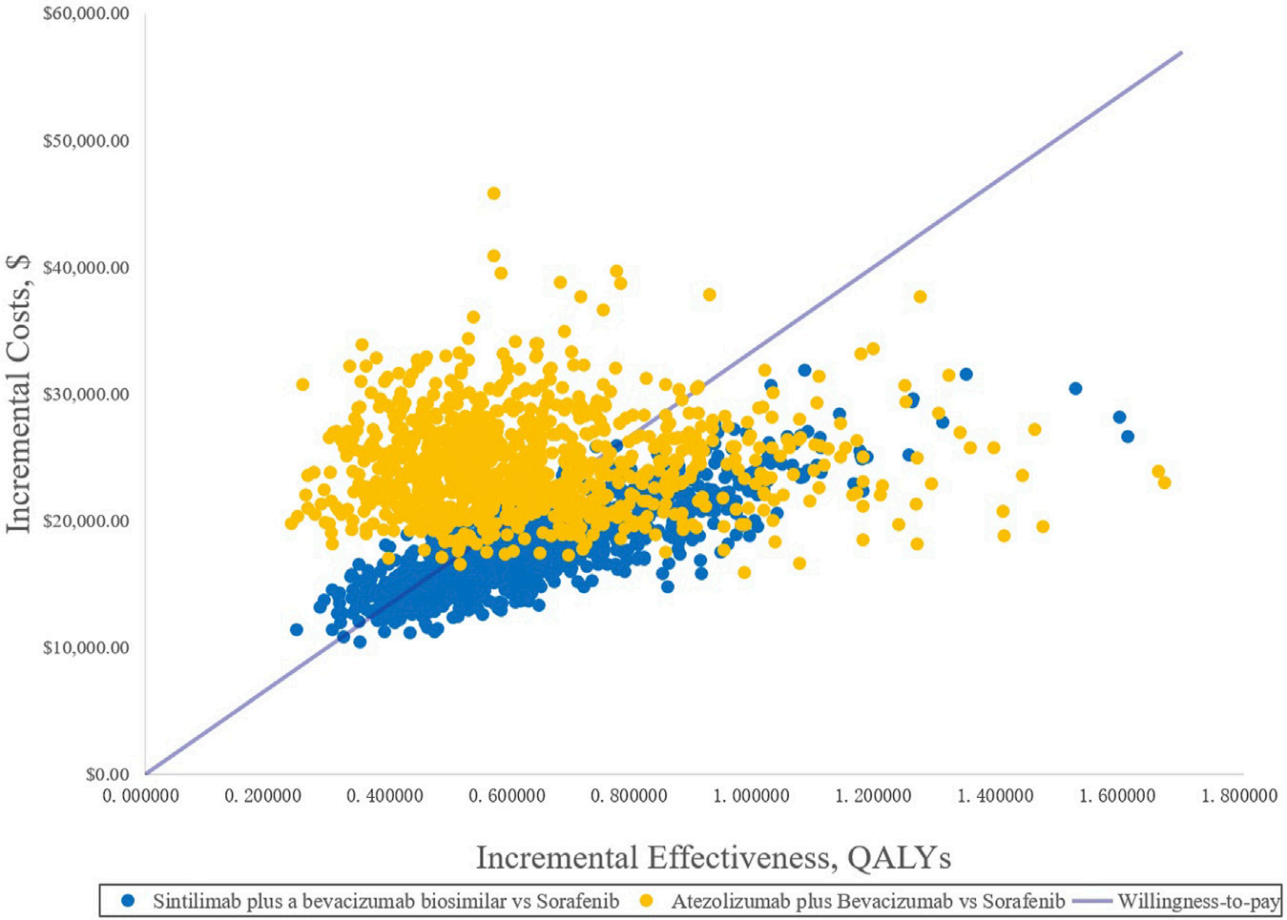
Incremental cost-effectiveness scatterplots of the scenario analysis. QALYs, quality-adjusted life years.

Because of the high price of PD-1 and PD-L1, they are not affordable for many patients in China; therefore, sintilimab and atezolizumab PAP were implemented for Chinese patients. The specific scheme is described in the methodology (2.3.2). The one-way DSA of scenario analysis revealed that the HRs of the OS of both SB and AB regimens against sorafenib were the most sensitive parameters, and the ones which had the most prominent influence on the ICERs. When SB versus sorafenib, the results were also sensitive to the price of the bevacizumab biosimilar and the proportion of subsequent therapy in the SB regimen, while the HRs of PFS and the price of atezolizumab were sensitive when AB versus sorafenib. The results are shown in the tornado diagram in Figure 5. The CEAC of the scenario analysis (Figure 6) showed that the likelihood of SB and AB regimens being cost-effective was 76.2 and 30.4%, respectively, compared with sorafenib at a WTP threshold of $33,500/QALY. The incremental cost-effectiveness scatterplot is shown in Figure 7.

## 4 Discussion

Two phase III clinical trials (ORIENT-32 and IMbrave150) revealed a survival advantage of ICIs plus anti-VEGF drugs compared with the standard treatment (sorafenib) for unresectable HCC (Finn et al., 2020a; Ren et al., 2021). At present, there has been no head-to-head clinical trial of SB and AB for the treatment of unresectable HCC. Therefore, in this study, the two treatments were indirectly compared by a network meta-analysis; in addition, a cost-effectiveness comparison between the regimes was conducted. To the best of our knowledge, this is the first study to explore the cost-effectiveness of SB and AB compared with sorafenib for the treatment of unresectable HCC.

In this study, we adopt a partitioned survival model. Before selecting the model, we looked for pharmaco-economic literature and found that more researchers have used the Markov model for unresectable HCC (Zhang et al., 2016; Cai et al., 2020; Wen et al., 2021). However, the Markov model needs to assume and estimate the transition probability. A partitioned survival model does not need to calculate the transition probability; it can be directly derived from the partitioned survival model, which is simpler and easier to calculate and is closer to the actual observed data (Hoyle et al., 2011). The partitioned survival model has been increasingly applied to the pharmaco-economic evaluation of advanced cancer treatments.

Considering the rising medical costs, value-based oncology is worthy of our attention. SB and AB are the leading therapies in the immunotherapy pipeline and have received considerable attention. Our study found that compared with sorafenib, SB improved the effectiveness by 0.617 QALYs, resulting in an ICER of $39,766.86/QALY, and the treatment of AB produced an additional 0.596 QALYs compared with sorafenib, resulting in an ICER of $103,037.66. The ICERs of both SB and AB, compared with sorafenib, exceeded the WTP threshold ($33,500/QALY). In the scenario analysis, we considered PAP, and found that the ICER of SB versus sorafenib ($28,539.82/QALY) was lower than the WTP threshold ($33,500/QALY). However, the ICER of AB versus sorafenib ($40,524.30/QALY) was still higher than the WTP when considering PAP.

In the IMbrave150 trial, compared with sorafenib, AB had a significant effect in patients with unresectable HCC without systemic treatment. However, many scholars have carried out a pharmaco-economic evaluation of AB in the treatment of unresectable HCC, and most of the findings were similar to ours, and showed that AB was not a cost-effective first-line choice for unresectable HCC; however, extreme cost-cutting may change the results (Hou and Wu, 2020; Wen et al., 2021). In the ORIENT-32 trial, SB showed a significant OS and PFS benefit in patients with unresectable HCC. Through the analysis of HR for OS and PFS by network meta-analysis, we found that SB exhibits a slight advantage over AB in terms of curative effect. In terms of cost, the cost of SB is relatively low; therefore, SB is a cost-effective therapeutic regimen if PAP is considered.

Although both are ICIs, there was a huge gap in the cost between sintilimab and atezolizumab because of the following reasons: first, by considering the affordability of Chinese patients, the first price of sintilimab is relatively lower than atezolizumab because sintilimab is first approved by the Chinese government. Second, high reimbursed prices for new cancer medicines, certainly in Europe, have been enhanced by the emotive nature of cancer (Haycox, 2016; Godman et al., 2021). Meanwhile, the notion is that the US federal government is prohibited by law from negotiating drug prices as a result of the 2003 Medicare Prescription Drug, Improvement and Modernization Act (Workman et al., 2017). In addition, there can be high profitability for new cancer medicines as seen before they lose their patents (Godman et al., 2019). Therefore, the high requested/expected prices for new medicines for cancer and orphan diseases mean these two areas dominate new medicines being researched (Global, 2022). The sensitivity analysis also showed that the cost of atezolizumab had a significant impact on the model results, which led to the cost-effectiveness results in China. Thus, when the unit cost of atezolizumab decreased by 80%, the ICER for AB decreased to close to $33,500/QALY.

Our study has some limitations. First, the populations selected in the two RCTs were different: the ORIENT-32 trial recruited participants from the Chinese population and the IMbrave150 trial recruited globally. The survival information of patients by nationality was not presented in the RCT results. Moreover, owing to the lack of head-to-head experimental data, the network meta-analysis could not perform an inconsistency test, so the data might be biased. Second, because SB was approved only in China, the results of this study should be carefully explained when the results are transferred to other regions. Third, our study only included the costs and disutilities of grade three or four AEs, and ignored the costs and disutilities of AEs below grade 3. Fourth, this study extracted the utility values of PFS and PD status from the published literature, which will affect the arithmetic of the clinical efficacy. Fifth, the IPD used in our model was simulated using the algorithm recommended by Guyot et al. (2012). It is generated by time-event data, which deviate slightly from the actual individual patient data. Finally, we did not check the economic outcomes in subgroups, such as the age of the patients, which may have an impact on the results.

## 5 Conclusion

In summary, the findings from our study showed that sintilimab + a bevacizumab biosimilar is a cost-effective regimen compared with sorafenib as the first-line therapy for unresectable HCC in China at a $33,500 WTP threshold if sintilimab PAP was considered. However, the atezolizumab + bevacizumab regimen is not a cost-effective tactic, regardless of whether atezolizumab PAP is considered.

## Data Availability

The original contributions presented in the study are included in the article/Supplementary Material; further inquiries can be directed to the corresponding authors.
