# Supplementary figures and images for: Immune Checkpoint Inhibitors Plus an Anti-VEGF Antibody as the First-Line Treatment for Unresectable Hepatocellular Carcinoma: A Network Meta-Analysis and Cost-Effectiveness Analysis
Source: Front Pharmacol. 2022 Jun 1;13:891008. doi: 10.3389/fphar.2022.891008 (PMC9198580; doi:10.3389/fphar.2022.891008)

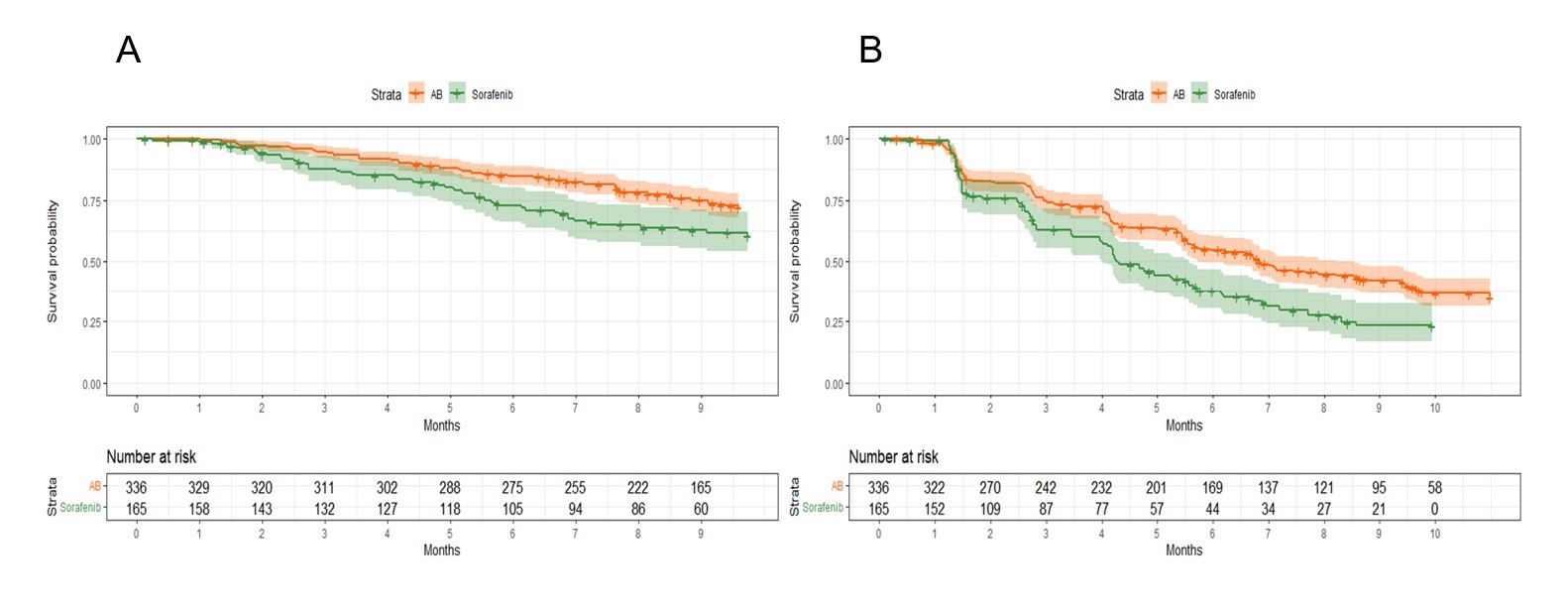

Supplement: Supplementary file 1 [file Image3.JPEG]

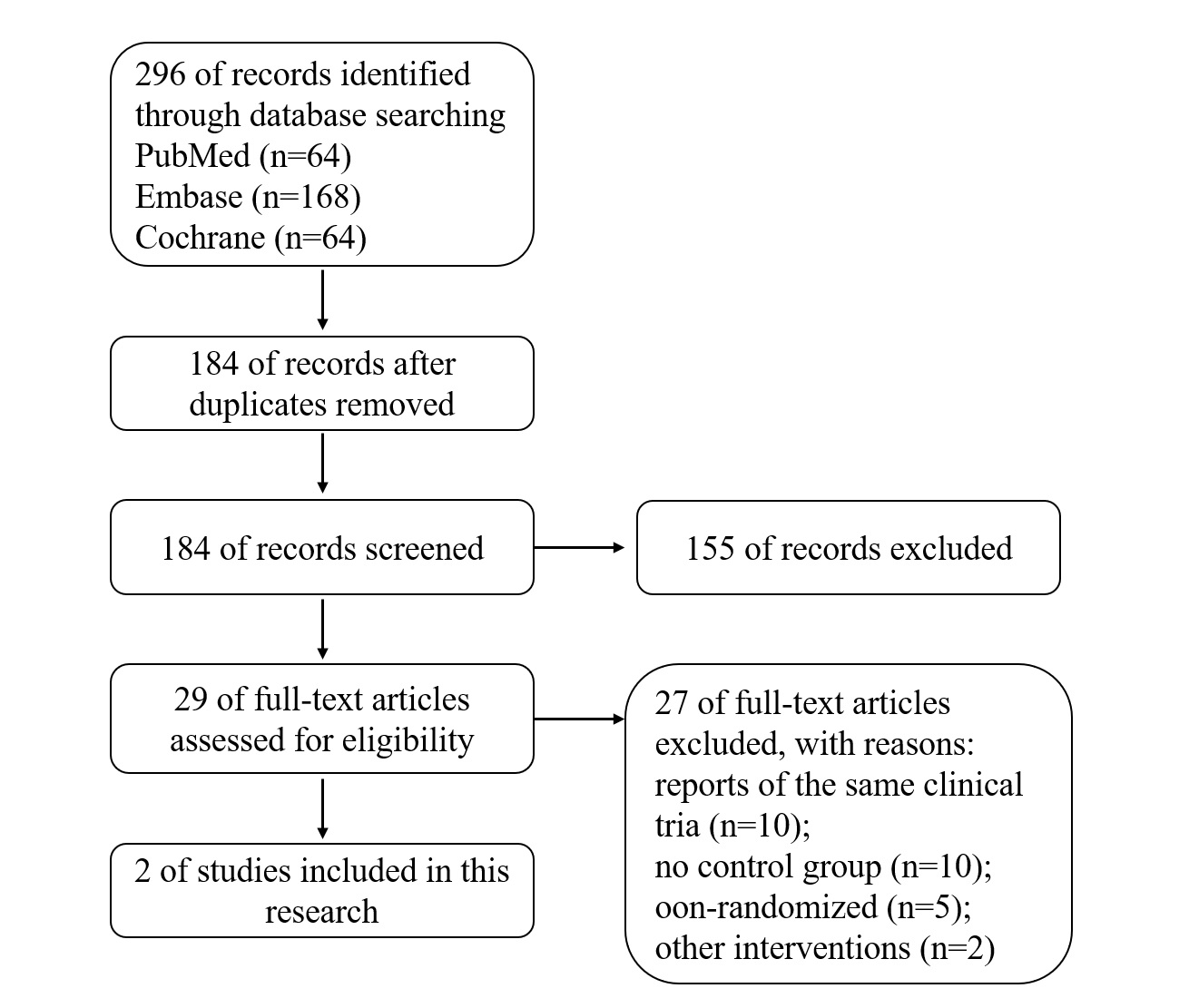

Supplement: Supplementary file 2 [file Image1.JPEG]

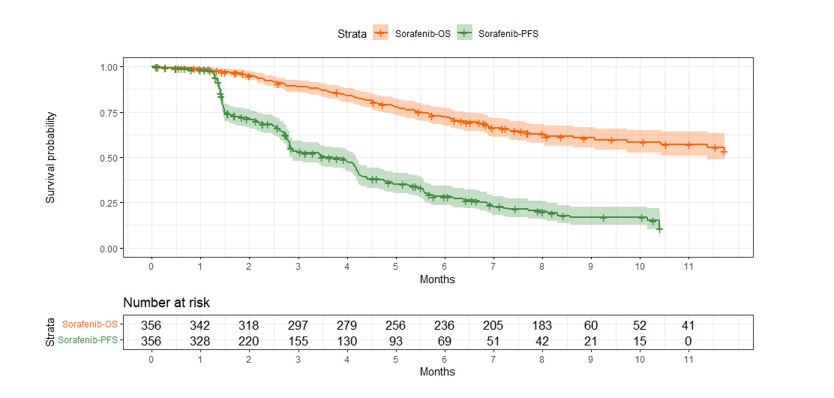

Supplement: Supplementary file 3 [file Image4.JPEG]

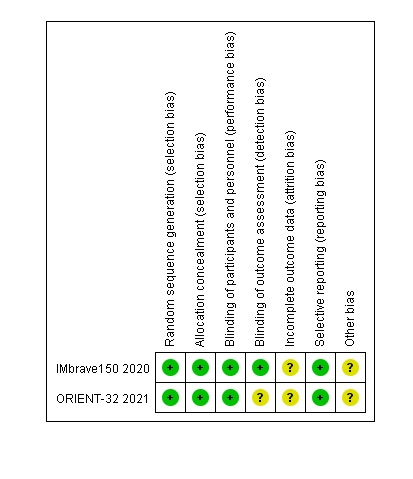

Supplement: Supplementary file 4 [file Image7.JPEG]

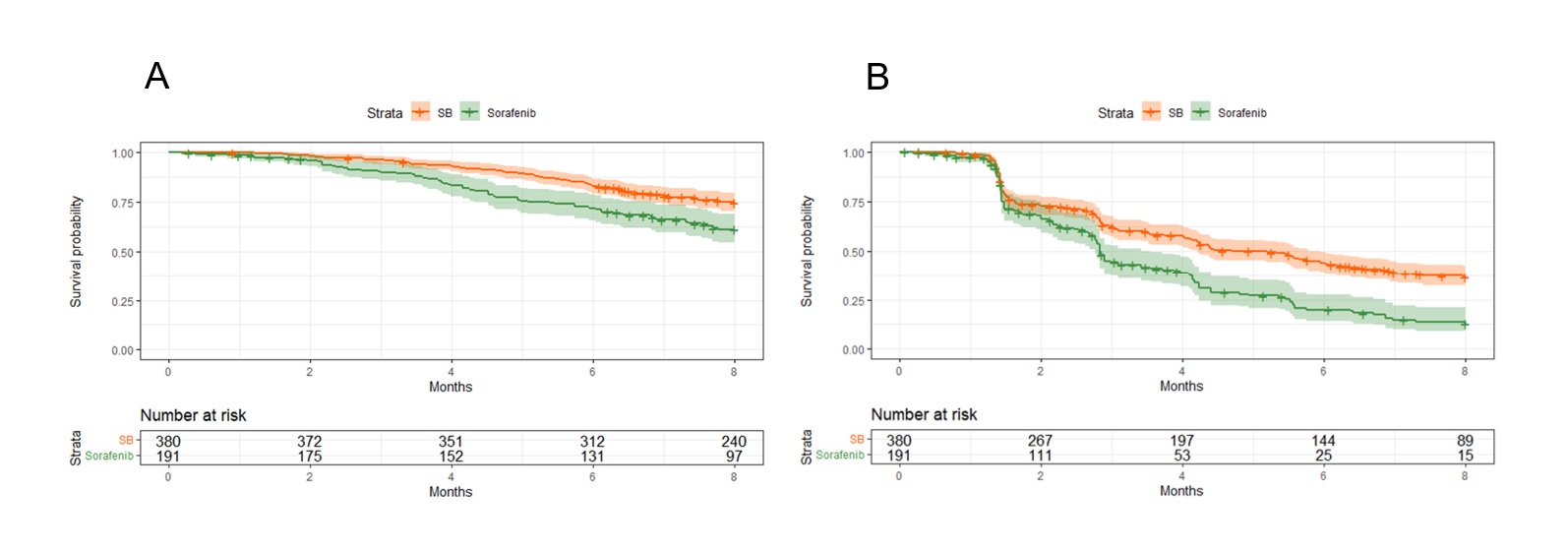

Supplement: Supplementary file 5 [file Image2.JPEG]

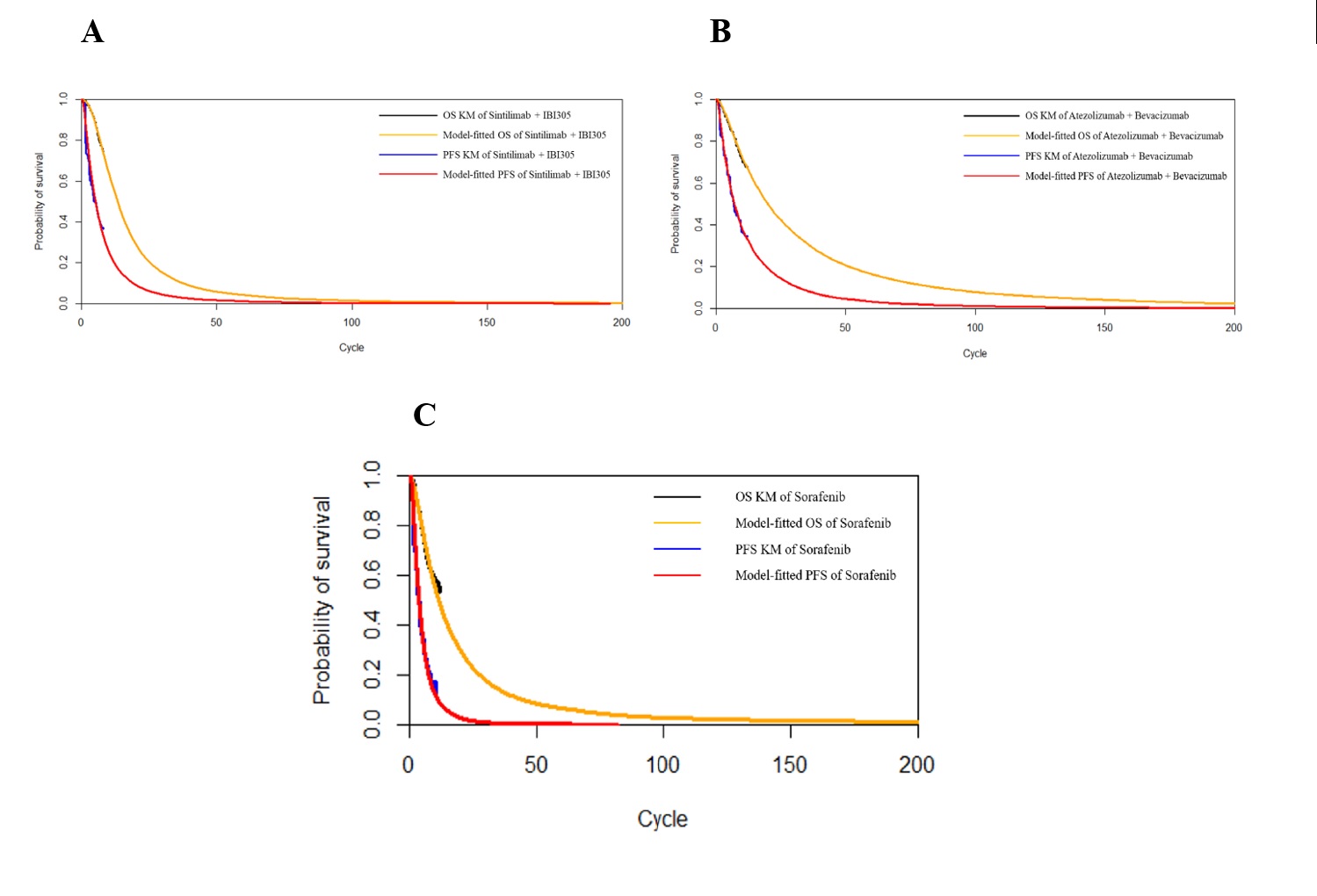

Supplement: Supplementary file 6 [file Image5.JPEG]

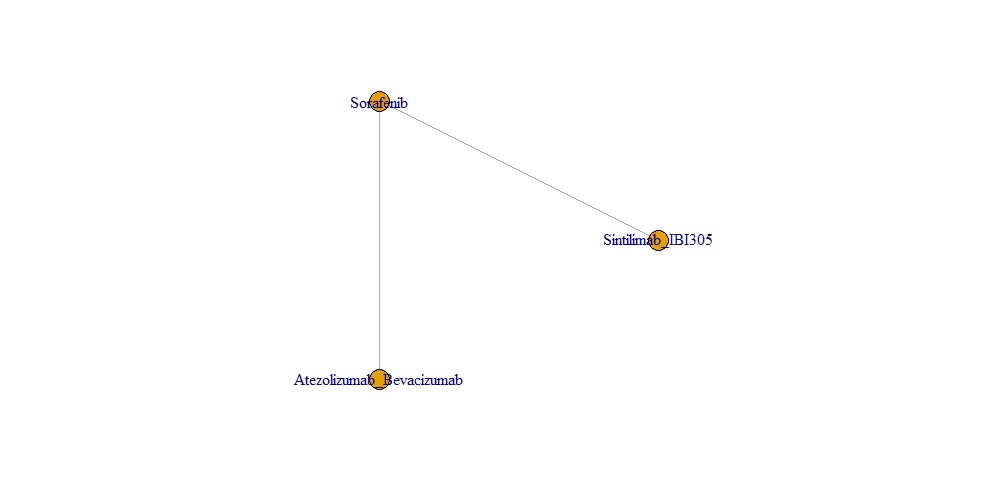

Supplement: Supplementary file 8 [file Image6.JPEG]
