# Supplementary material for: Immune Checkpoint Inhibitors Plus an Anti-VEGF Antibody as the First-Line Treatment for Unresectable Hepatocellular Carcinoma: A Network Meta-Analysis and Cost-Effectiveness Analysis
Source: Front Pharmacol. 2022 Jun 1;13:891008. doi: 10.3389/fphar.2022.891008 (PMC9198580; doi:10.3389/fphar.2022.891008)
Supplement: Supplementary file 7 [file DataSheet1.docx]

Supplementary Material

**1 Supplementary Figures and Tables**

- 1. **Supplementary Figures**

**
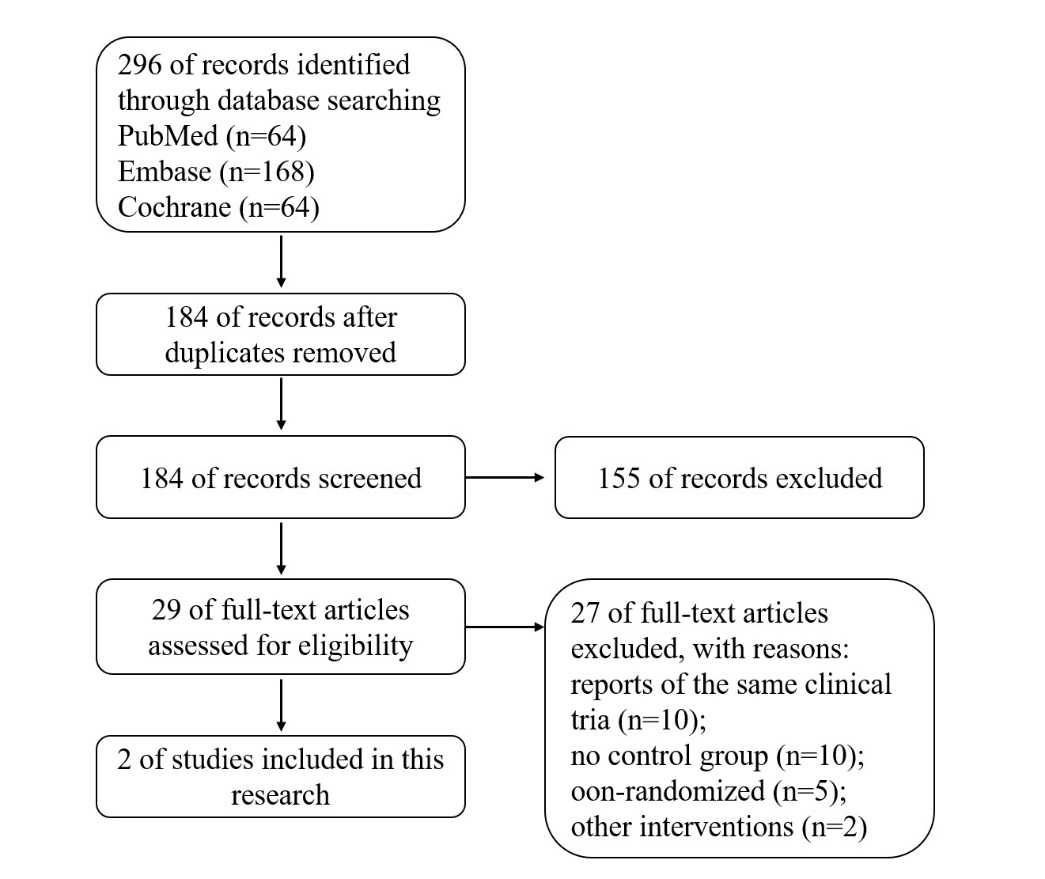
**

**Supplementary Figure 1** Flowchart of Study Selection


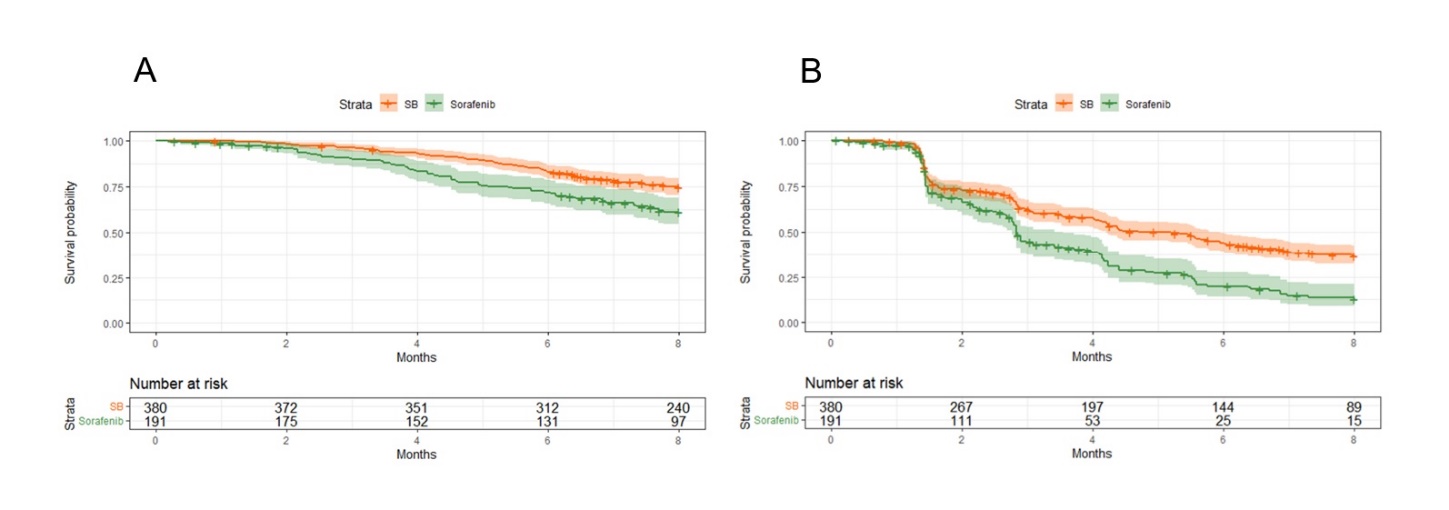


**Supplementary Figure 2** The replicated KM OS (A) and PFS (B) curves of SB (orange) and Sorafenib treatments (green) in ORIENT-32 trial.

Abbreviations: OS, overall survival; PFS, progression-free survival; SB, sintilimab plus a bevacizumab biosimilar (IBI305); KM, Kaplan-Meier.


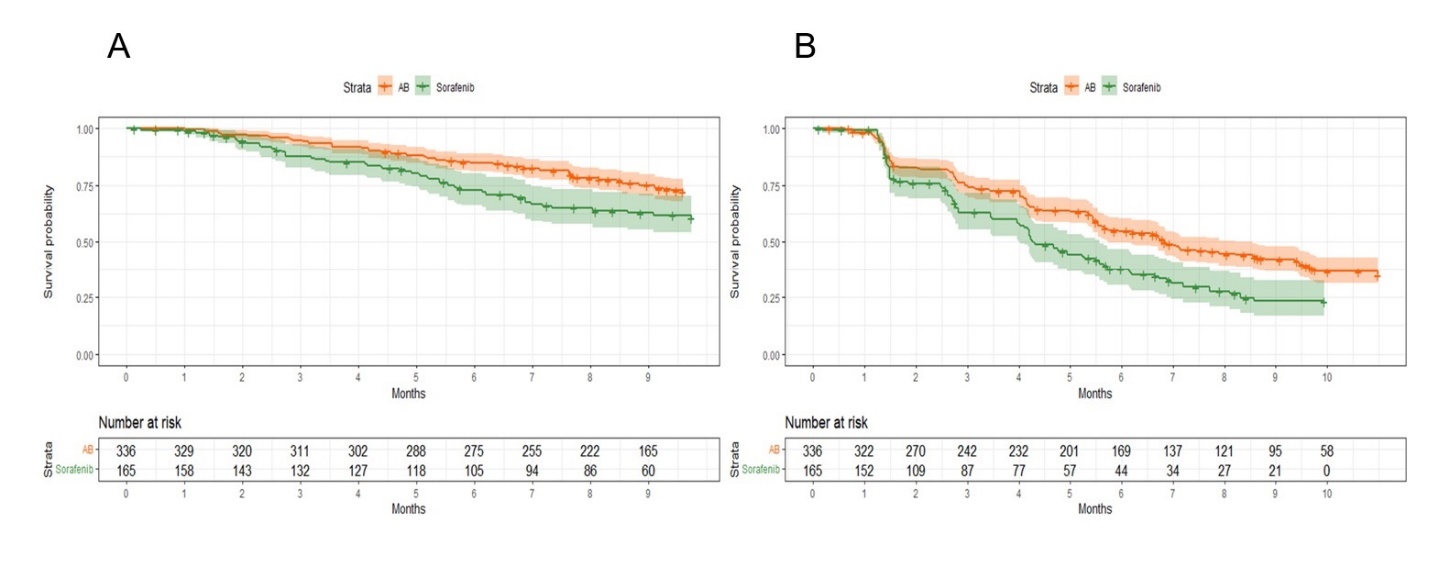


**Supplementary Figure 3** The replicated KM OS (A) and PFS (B) curves of AB (orange) and Sorafenib treatments (green) in IMbrave150 trial.

Abbreviations: OS, overall survival; PFS, progression-free survival; AB, atezolizumab plus bevacizumab; KM, Kaplan-Meier.


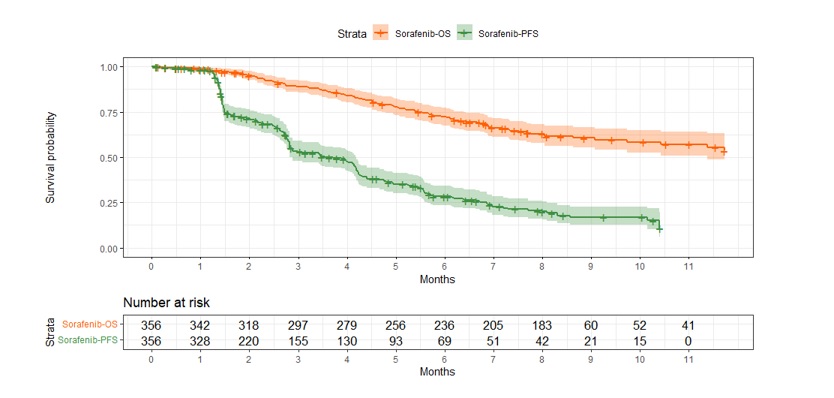


**Supplementary Figure 4** The replicated KM OS (orange) and PFS (green) curves of sorafenib treatment by pooling the ORIENT-32 and IMbrave150 trialtrials.

Abbreviations: OS, overall survival; PFS, progression-free survival; KM, Kaplan-Meier.


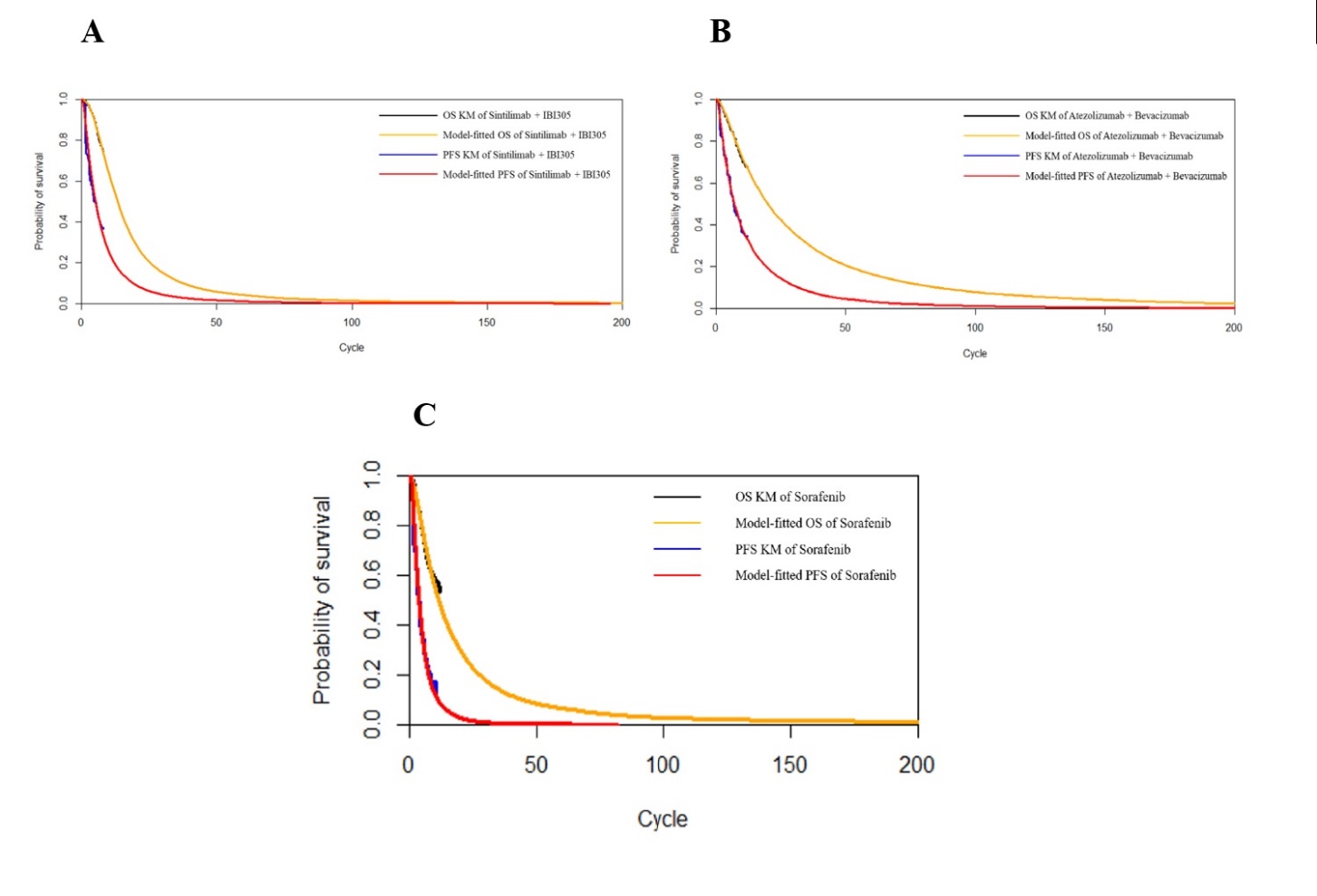


**Supplementary Figure 5** Original versus model-fitted Kaplan-Meier curves. The solid orange lines represent the modeled survival curves of the OS of the three regimens, the solid red lines represent the modeled survival curves of the PFS of the three regimens and the black and blue lines are the actual survival curves. (A) Actual and modeled OS and PFS curve of SB; (B) Actual and modeled OS and PFS curve of AB. (C) Actual and modeled OS and PFS curve of sorafenib by pooling the ORIENT-32 and IMbrave150 trials. Each cycle of the x-axis is one month.

Abbreviations: OS, overall survival; PFS, progression-free survival; KM, Kaplan-Meier; SB, sintilimab plus a bevacizumab biosimilar (IBI305); AB, atezolizumab plus bevacizumab.

**
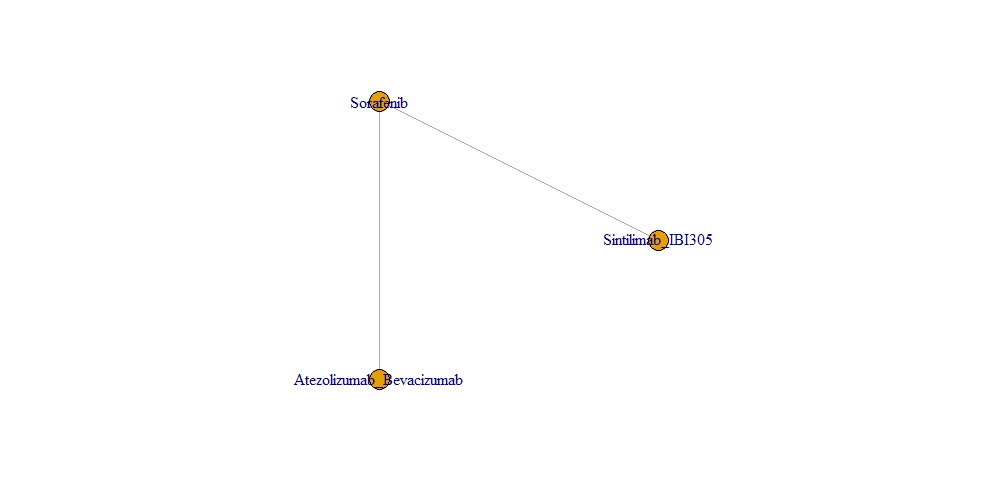
**

**Supplementary Figure 6** Model Schematic for Network Meta-analysis

**
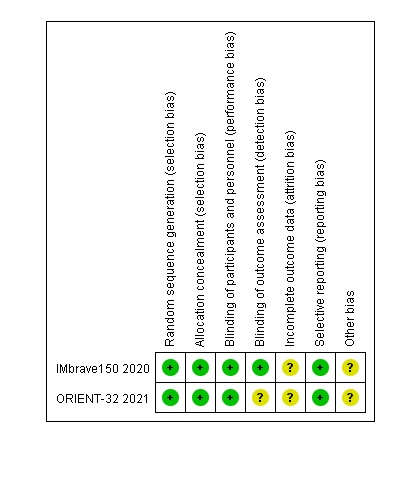
**

**Supplementary Figure 7** Risk of Bias Summary

- 1. **Supplementary Tables**

**Supplementary Table 1** Patient Baseline Characteristics.

| **Characteristic** | **SB** | **AB** |
| --- | --- | --- |
| Median age (range), years | 53 (21–82) | 64 (56–71) |
| Male sex | 88% | 82% |
| α-fetoprotein |  |  |
| ＜400ng/mL | 57% | 62% |
| ≥400ng/mL | 43% | 38% |
| ECOG performance status |  |  |
| 0 | 48% | 62% |
| 1 | 52% | 38% |
| Barcelona Clinic Liver Cancer stage |  |  |
| B | 15% | 15% |
| C | 85% | 82% |
| Macrovascular invasion | 28% | 38% |
| Extrahepatic metastasis | 73% | 63% |
| Cause of hepatocellular carcinoma |  |  |
| Hepatitis B | 94% | 49% |
| Hepatitis C | 2% | 21% |
| Abbreviations: ECOG, Eastern Cooperative Oncology Group; SB, sintilimab plus a bevacizumab biosimilar (IBI305); AB, atezolizumab plus bevacizumab. | | |

**Supplementary table 2** Parameters of parametric models for virtual time-to-event data.

| Trial names | Treatment regimens | Endpoint | Distribution | Distribution information | AIC |
| --- | --- | --- | --- | --- | --- |
| ORIENT-32 | Sorafenib | OS | Log-logistic | shape:1.727 scale:10.345 | 528.7496 |
|  |  | PFS | Log-normal | meanlog:1.1217 sdlog:0.7770 | 620.66 |
|  | SB | OS | Log-logistic | shape:2.139 scale:13.086 | 783.5951 |
|  |  | PFS | Log-normal | meanlog:1.6041 sdlog:1.0377 | 1301.563 |
| IMbrave 150 | Sorafenib | OS | Log-logistic | shape:1.48 scale:12.62 | 460.54 |
|  |  | PFS | Log-normal | meanlog:1.484 sdlog:0.9098 | 565.774 |
|  | AB | OS | Log-normal | meanlog:2.9253 sdlog:1.1150 | 738.7586 |
|  |  | PFS | Log-normal | meanlog:1.9188 sdlog:1.0977 | 1246.48 |
| Pooling data of ORIENT-32 and IMbrave 150 trials | Sorafenib | OS | Log-logistic | shape:1.577 scale:11.477 | 1004.024 |
|  |  | PFS | Log-normal | meanlog:1.2942 sdlog:0.8621 | 1213.427 |
| Abbreviations: AIC, Akaike information criterion; OS, Overall survival; PFS, Progression-free survival; SB, sintilimab plus a bevacizumab biosimilar (IBI305); AB, atezolizumab plus bevacizumab. | | | | | |
